# Statistical optimization of amorphous iron phosphate: inorganic sol–gel synthesis-sodium potential insertion

**DOI:** 10.1186/s13065-021-00774-x

**Published:** 2021-08-17

**Authors:** Fz. Maarouf, S. Saoiabi, K. Azzaoui, C. Chrika, H. Khalil, S. Elkaouni, S. Lhimr, O. Boubker, B. Hammouti, S. Jodeh

**Affiliations:** 1 Laboratoire de Chimie Appliquée Des Matériaux (LCAM), Faculty of Sciences, Mohammed V University, Rabat, Morocco; 2 LCAE-URAC18, COST, Department of Chemistry, Faculty of Sciences, Mohamed 1st University, P.O. Box 717, 60000 Oujda, Morocco; 3 Management and IS Research Group - National School of Business & Management, Abdelmalek Essaadi University, Tangier, Morocco; 4 Department of Chemistry, College of Science, An-Najah National University, Nablus, Palestine; 5 Department of Management, Laayoune Higher School of Technology, Ibn Zohr University, Agadir, Morocco

**Keywords:** Amorphous, Iron phosphate, Inorganic sol–gel, IEP, PZC, Surface property, Surface charge, Statistical optimization, Sodium insertion

## Abstract

Iron phosphate, Fe_2_ (HPO_4_)_3_*4H_2_O, is synthesized at ambient temperature, using the inorganic sol–gel method coupled to the microwave route. The experimental conditions for the gelling of Fe (III)-H_3_PO_4_ system are previously defined. Potentiometric Time Titration (PTT) and Potentiometric Mass Titration (PMT) investigate the acid–base surface chemistry of obtained phosphate. Variations of surface charge with the contact time, Q a function of T, are examined for time contact varying in the range 0–72 h. The mass suspensions used for this purpose are 0.75, 1.25 and 2.5 g L^−1^. The point of zero charge (PZC) and isoelectric point (IEP) are defined using the derivative method examining the variations $\frac{{{\text{dpH}}}}{{{\text{d}}t}} = f\left( {{\text{pH}}} \right)$, at lower contact time. A shift is observed for PZC and IEP towards low values that are found to be 2.2 ± 0.2 and 1.8 ± 0.1, respectively. In acidic conditions, the surface charge behavior of synthesized phosphate is dominated by $\overline{{ > {\text{POH}}}}$ group which pK_a_ = 2.45 ± 0.15. Q against T titration method is performed for synthesized Fe_2_ (HPO_4_)_3_*4H_2_O in NaCl electrolytes. The maximal surface charge (Q) is achieved at the low solid suspension. Hence, for m = 0.75 g L^−1^, Q value of 50 coulombs is carried at μ = 0.1 and pH around 12, while charge value around 22 coulombs is reached in the pH range: 3–10. The effect of activation time, Q and pH on sodium insertion in iron phosphate, were fully evaluated. To determine the optimal conditions of the studied process, mathematical models are used develop response surfaces in order to characterize the most significant sodium interactions according to the variation of the pH, Q, the contact time and the contents of the synthesized material.

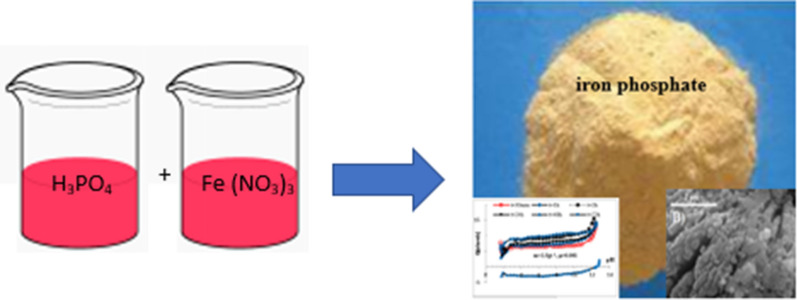

## Introduction

The emerging attention to environmental pollution and energy consumption and the rapid promotion and innovation of electronic devices has created a surge in demand for more efficient and clean energy storage and conversion devices. To avoid the use of rare metals, extensive research is undertaken to develop a second generation of batteries, known as post-lithium ion secondary batteries. Sodium and potassium are among the elements proposed as charge carriers in these batteries. The large ionic dimension of Na^+^ (1.02 Å) compared to Li^+^ (0.76 Å) is coupled to slow diffusion, interphase formation and phase stability [1, 2]. The storage capacities in crystalline hosts are limited by the low ion migration associated to the stoichiometric restraint of ion insertion. Compared to lithium insertion, the incorporation of Na^+^ and K^+^ ions is considered as big challenge, in this material. The main diffusion mechanism involving a single step is performed in one-dimensional channels. To overcome this phenomenon requires appropriate electrode active materials [3, 4]. Thus, open framework materials such as phosphates have emerged as host matrices for these alkaline elements intercalation/deintercalation [5]. The amorphous products with disordered structures may electrochemically store and release these voluminous alkali ions. For this purpose, amorphous iron phosphates which are chemically stable are considerably explored for use in sodium ion batteries. So, NaFePO_4_ is a promising cathode material for no-lithium batteries. Due to the high activation barriers, Na^+^ diffusion is of lower extent in crystalline Fe-phosphates. As a result, Na^+^ extraction from these electrode materials requires an activation energy lowering that is achieved through amorphization of phosphates and results in an increase of Na diffusion [6]. Little is known about this diffusion process which is governing the phase stability of Na_x_FePO_4_ (x < 1) during sodiation–desodiation phenomenon. The insertion chemistry of lithium and sodium carried out in FePO_4_ are, therefore, considerably different [4]. Hence, the development of the post-lithium batteries requires to understand several processes of sodium and potassium ion chemistry and surface chemistry such as kinetics and insertion mechanisms of Na(K)FePO_4_ electrodes [7].

One can note that although the amorphous state of FePO_4_ is suitable for electrochemical activity, a pre- sodiation (or potassiumation) step is crucial and can be performed by chemical or electrochemical ways. The surface properties and the electrified hydrated interfaces play a main role in interaction of phosphate aggregates with various aqueous particles. The surface suspensions charge occurs from proton transfer during ionization (protonation and deprotonation) of the surface hydroxyl groups, > S–OH, which results in > SOH_2_^+^ or > SO^−^ species. Generally it is admitted that ionization reactions are controlled, essentially, by the pH and ionic strength of dispersion medium.

It is shown previously that the increase in mass of some metal suspensions is associated with an increase in point of zero charges (PZS) value. This increase is suggested to be a result of oxide surface hydration. The value of the isoelectric point (IEP) is dependent on initial pH and decreases as this pH and electrolyte salt (KNO_3_) concentration increase [8]. The proton affinity of surface groups is then essential in H^+^/OH^−^ sorption exchange processes and it determines the charging and the binding behavior of sorbents [9]. In general, the point of zero charges is identified as the initial pH corresponding to minimal variation and associated with ΔpH ~ 0. Also, it is assumed that surface equilibrium is reached after a contact time of about 24 h [10].

In this study, acid iron phosphates are synthesized following a sol–gel route associated to a microwave irradiation (800w) for an about 15 s. The synthesis was undertaken within the pH formation range of monohydrogen phosphate. The surface chemistry of the so-obtained particles was studied to understand the Fe/P ratio effects. To achieve this purpose, two potentiometric techniques were carried out using Potentiometric Time Titration (PTT) and Potentiometric Mass Titration (PMT). The suspensions used, for this purpose, were of 0.75, 1.25 and 2.5 g/L. These suspensions were aging for different times (T) up to 72 h.

## Materials and methods

All chemicals are of reagent quality (Sigma-Aldrich) and used without purification. Phosphoric acid (0.1 M) and iron (III) nitrate (0.1 M) were used as the starting materials. The ionic strength, µ, is adjusted to value of 0.001, 0.01 and 0.1 with NaCl as electrolyte salts.

The batch equilibration technique was applied to determine the Point of Zero Charge (PZC). To achieve this purpose, titrations of HNO_3_ (0.05 M)-iron phosphate systems were carried out at room temperature with KOH (0.05 M). Surface charge variations Q against T, were examined for FePO_4_ suspensions of 0.75, 1.25 and 2.5 g/L.

### Synthesis of iron (III) phosphate

Iron (III) phosphate was synthesized by a modified inorganic sol–gel method [11]. A solution of a Fe/P molar ratio of 1:1.5 is gellified by a microwave irradiation and the resulting gel is oven-dried at 50 °C for about 48 h. The xerogels are washed with distilled water and ethanol to obtain the final phosphates. All the phosphate samples were then characterized using X-ray diffraction analysis and FTIR spectroscopy. The results show that the amorphous phase was Fe_2_ (HPO_4_)_3_* xH_2_O.

### Experimental instruments

All the phosphate samples were then characterized using X-ray diffraction analysis and FTIR spectroscopy. The X-ray diffraction (XRD) spectra were performed using an X-ray powder diffractometer (LabxXRD-6100 Shimadzu) with an acquisition rate of 0.05 to 25°/min (θ)). The recording of our IR spectra in the 4000–250 cm^−1^ range is carried out using an IR spectrometer of the PERKIN ELMER 577 type or using an IR spectrometer 1600 FTIR, the samples studied are intimately crushed with KBr and shaped in the form of pellets. These techniques are used in conjunction with thermal analysis (DTA) and thermogravimetric analysis (TGA) to follow the thermal evolution of these Xerogel phosphates, Thermogravimetric (TGA) and differential thermal analysis (DTA) (Labsys Evo1F Setaram). Morphological surface features were examined by applying Jeol JSM-IT100 InTouchScope™ Scanning Electron Microscope (SEM) connected to a microanalyzer EDS [12].

## Results and discussions

### X ray diffraction

**Fig. 1:**
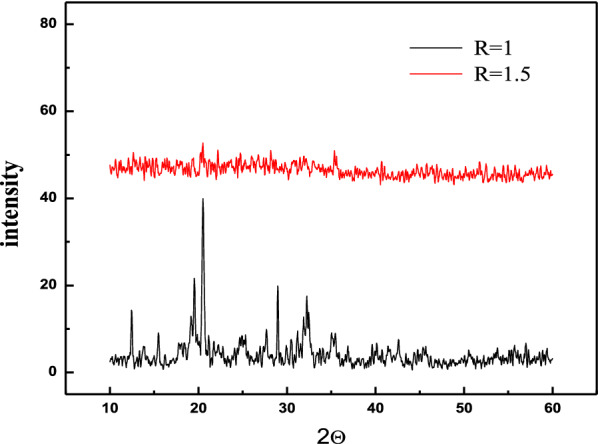
Diffractograms of xerogel obtained in Fe (III)-H_3_PO_4_ system with R = P/Fe = 1.5 and R = P/Fe = 1

Figure 1 (R = 1) shows the XRD spectrogram of iron phosphate. It can be seen that all three types of materials have sharp characteristic diffraction peak and the peak positions are very similar to each other. Compare with the JCPDS01-081-1173 standard spectrum diagram.

While at (R = 1.5) shows XRD patterns of synthesized iron phosphate powders. As shown, XRD patterns of as-synthesized phosphate shows no intense broad peaks indicating amorphous iron phosphate.

### Infrared spectroscopy

**Fig.2:**
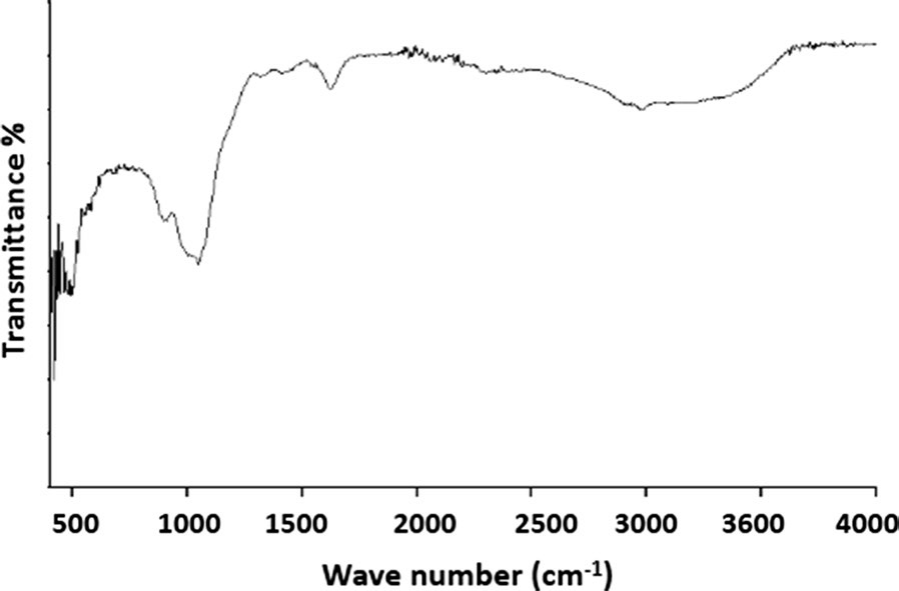
FTIR spectrum of iron phosphate with a ratio P/Fe = 1.5

The FTIR spectrum of synthesized iron phosphate is shown in Fig. 2. This FTIR spectrum is normalized to the intensity of the (PO) (P–O stretching band) at 1044 cm^−1^.

The functional groups of iron phosphate and precursor material are presented in Fig. 2. The presence of low intensity bands in the 3600–2700 cm^−1^ is characteristic of the hydroxyl-stretching vibrations of water, and acid phosphate. The IR band observed at 1640 cm^−1^ is associated with the strong hydrogen bonding of the water molecules to the phosphate anions. The large intense band at 1044 and the weak band at 900 cm^−1^ are associated with P–OH stretching vibrations of HPO_4_^2−^ [13–16]

The precursor sample also had symmetrical stretching vibration peak of P–O bond at 1428.66 cm^−1^, symmetrical stretching vibration peak of P–O bond at 526.13 and 1047.63 cm^−1^.

### DTA–TGA spectrum of the iron phosphate

**Fig. 3:**
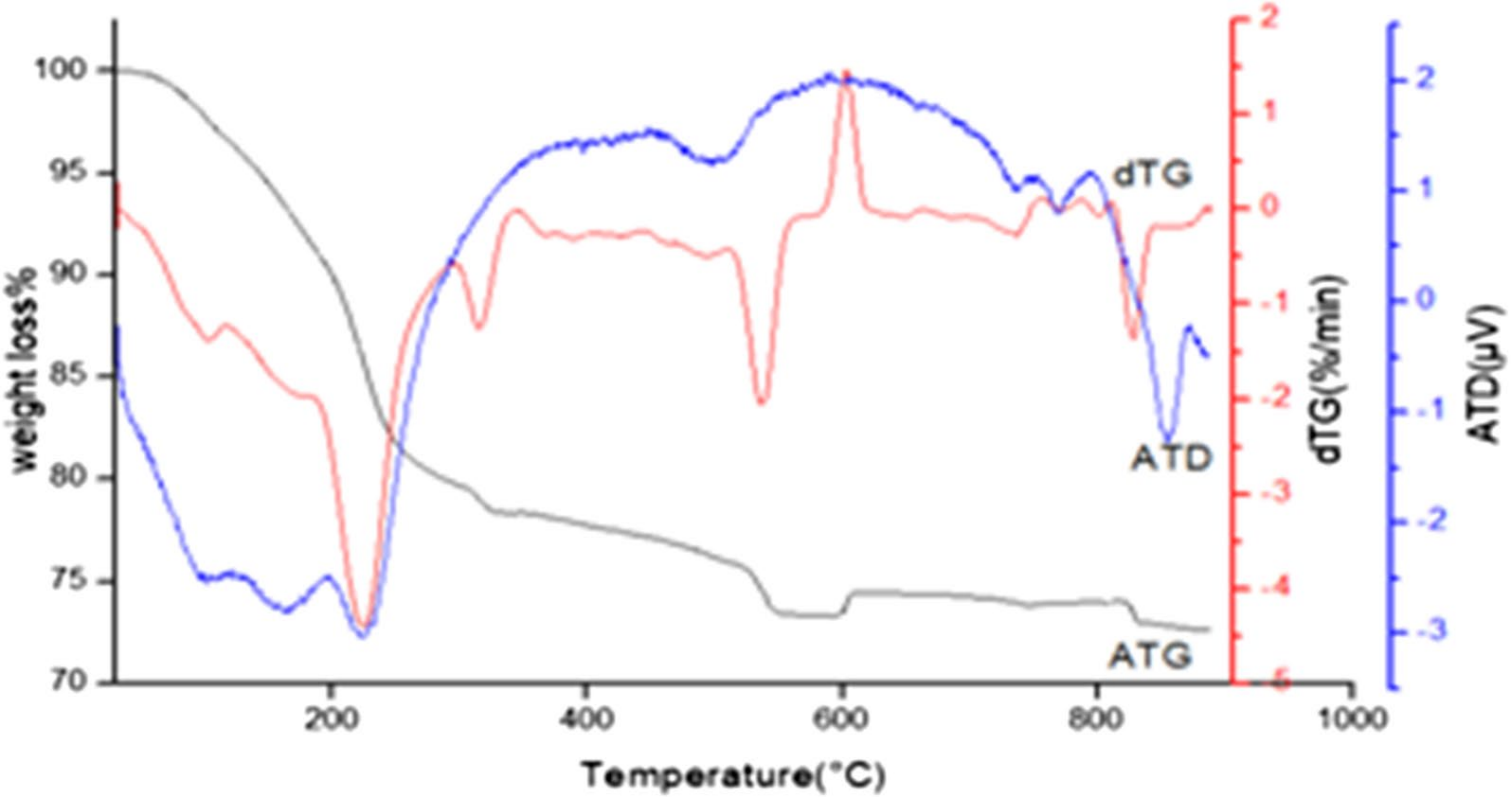
DTA–TGA of iron phosphate amorphous synthesized phosphate at P/Fe = 1.5

Thermal study of iron phosphates is carried out using thermogravimetric analysis (TGA) and differential analysis (DTA) from room temperature to about 900 °C. Obtained results are shown in Fig. 3.

The DTA curve indicates a sharp endothermic peaks appearing at 225, 370 and 540 °C. The first peak associated with weight loss of 15.1 wt.% on the TG curve is related to a quick dehydration of Fe_2_ (HPO_4_)_3_*4H_2_O. The second peak with low weight loss is due to initial decomposition involving dehydratation of phosphate matrix. The third peak represents the essential decomposition that is corresponding to the phosphate condensation in which a hydroxyl polymerized chain is obtained without structure destruction. Occurring polymerization process is associated to weight loss of about 1.4 wt.% [14, 18]. The obvious exothermic peak at 605 °C together with three tiny peaks at 762 °C, 795 °C, and endothermic peak at 815 °C, is attributed to the phase transformation. As shown previously, the tiny peaks are associated with three-step structural transformation of the iron phosphate framework [17, 18].

### Surface morphology of synthesized phosphate

**Fig. 4:**
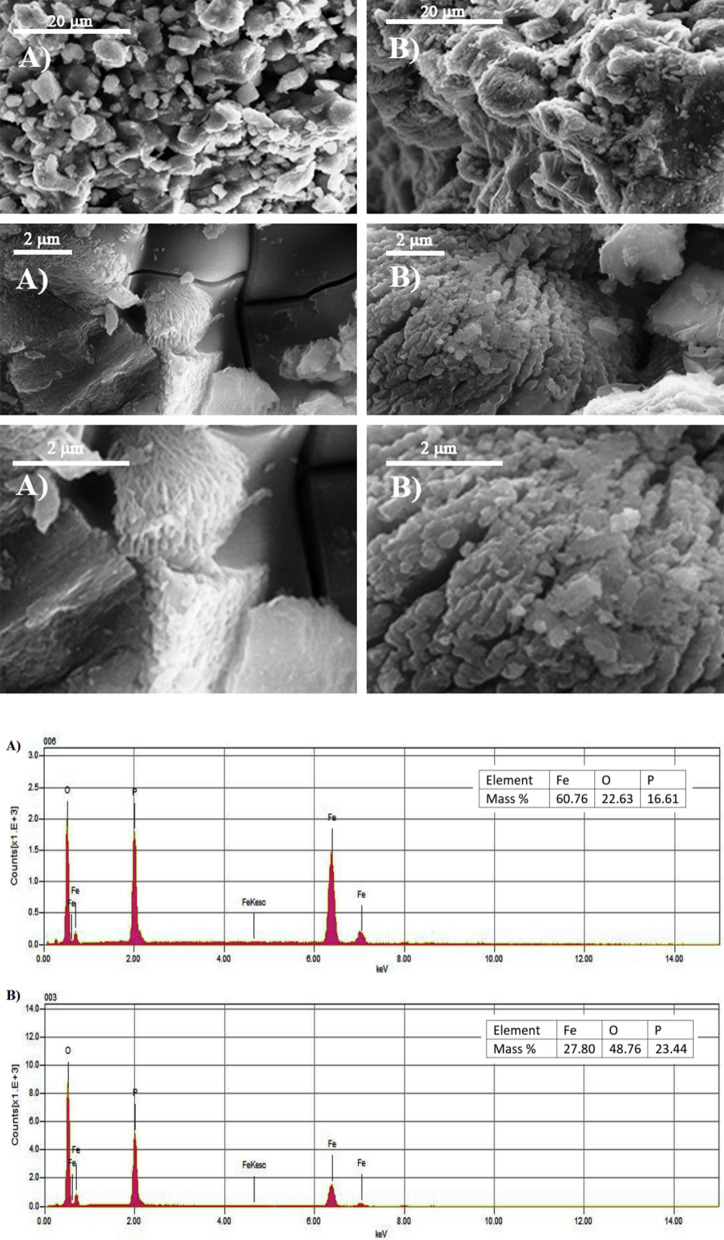
**A** SEM images and EDS results of as-prepared iron phosphate with Fe/P = 1.48. **B** SEM images and EDS results of as-prepared iron phosphate with P/Fe = 1.66

The surface morphology of synthesized phosphate samples is studied by the scanning electron microscopy (SEM) technique. Figure 4 shows the SEM micrograph and its energy dispersive X-ray spectroscopy (EDX) image obtained with 1000× magnification and a scale bar of 20 μm.

It is shown that the phosphate surface is porous and composed of Fe, P, and O, confirming the purity of phosphate materials. SEM studies show that agglomerates are plate and spherical in structure. The spherical aggregates are formed by cluster of small particles that are characterized by both low density and contact area. This result in higher specific surface is associated to greater porosity [19]. Consequently, spherical phosphates are more suitable for their electrochemical performance because of the Li ion diffusion [20].

### Point of zero charge

**Fig. 5:**
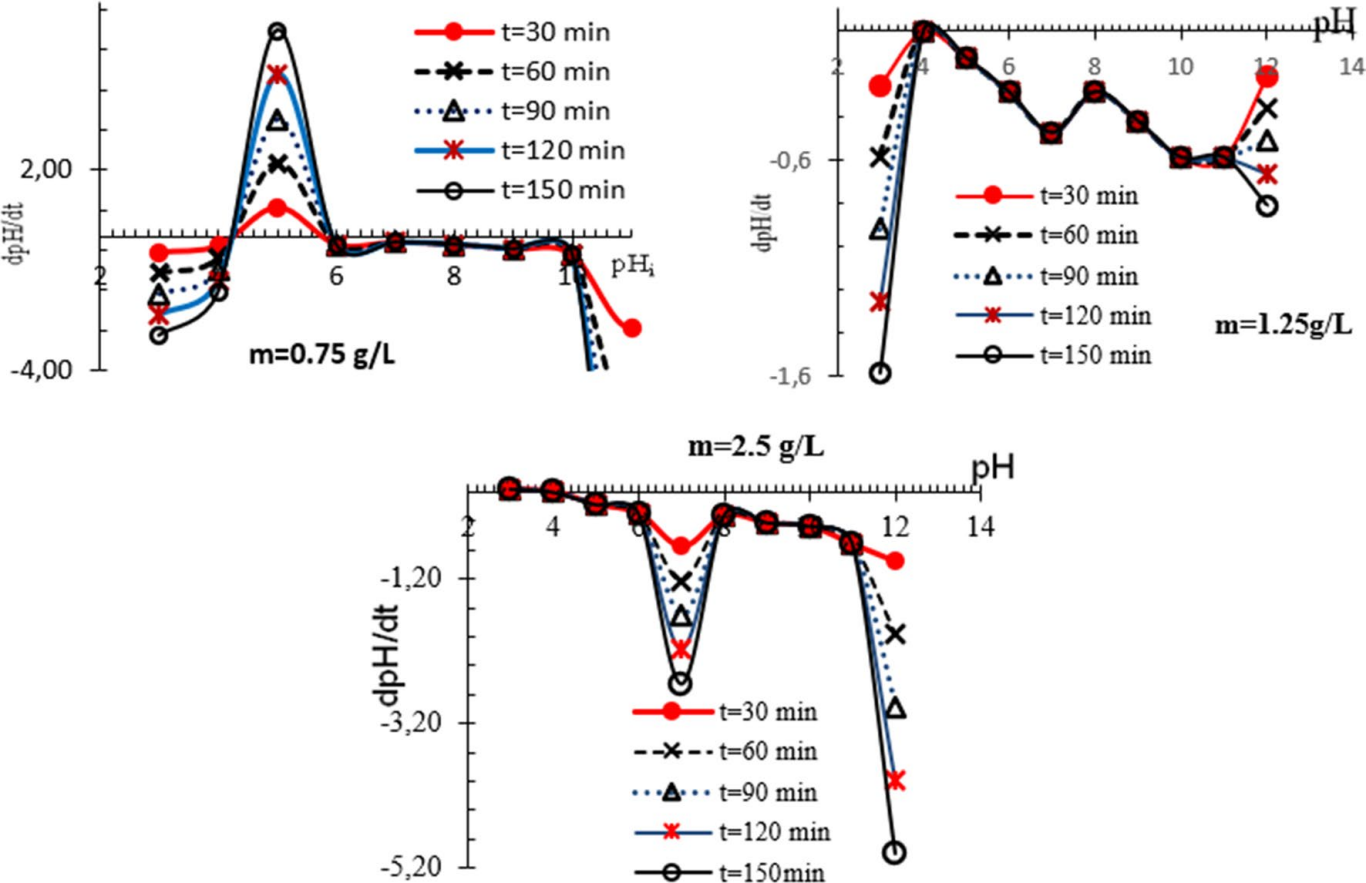
Variations of $\frac{{{\text{dpH}}}}{{{\text{d}}T}}\,in\, function\, of\, {\text{pH}}$ obtained for iron phosphate at m = 0.75, 1.25 and 2.5 g/L

In the present study, the point of zero charge (PZS) and isoelectric point (IEP) for ferric phosphate material are determined by kinetic-potentiometric method. The proton affinity which is of direct concern in proton exchange, is measured by pH change of phosphate suspensions. After agitation and settling time (T) varying from 30 to 150 min, the pH is measured for suspension of 0.75, 1.25 and 2.5 g/L. The initial pH is adjusted between 2 and 13, and the variations pH in function of T are best fitted (R^2^ > 99%) by the cubic equations. A first derivative method is used to graph $\frac{{{\text{dpH}}}}{{{\text{d}}T}}\,in\,function\, of\, {\text{pH}}$ shown in Fig. 5.

From obtained results, the surface reactions involve principally, proton release. The OH^−^ liberation or H^+^ uptake process is occurring at low suspension in a narrow pH range. It is important to note, as indicated previously, that the release of H^+^ to the solution results in deprotonated and negatively charged suspension [21].

As discussed previously, the pH response pattern is dependent on specific mass suspension. Thus, the rate of pH change observed at 1.25 and 2.5 g/L slurry is associated to proton liberation and results in pH decrease in the whole pH range. Vanishes of $\frac{{{\text{dpH}}}}{{{\text{d}}T}}$ with a broad maximum are occurring around pH = 4.0 and just correspond to zero charge, since no opposite in sign is observed for dpH. For 0.75 g/L suspension, rapid pH growth in the pH range 4–6 is crossing x-axis at the point of zero charge which value is 4.3 ± 0.1. A scarcity of literature data concerns the point of zero charge of iron(III) (hydrogen) phosphates, but it is important to note that obtained PZC value is higher, compared to that of Fe_3_(PO_4_)_2_, and Cr(III) and Al(III) phosphates which values are 3.3, and 2.6, respectively. For rare earth phosphates, the IEP and PZC values are found to occur in the pH range of 6.0 to 7.2 [22–24]. The zero rate change recorded for 0.75 g/L, at pH varying from 6 to10 means that the net total charge exchange is equal to 0. Consequently, proton release is controlled mainly by mass suspension and diminishes as this mass increases. Furthermore, obtained results show that IEP = PZC = 4.3 ± 0.1 suggesting no specific sorption of ions other than protons [25].

The zero surface charges occurring around PZC are independent of initial pH and are presenting no shift with contact time. The results also demonstrate that $\frac{{{\text{dpH}}}}{{{\text{d}}T}} \,in\, function\, of\, {\text{pH}}$ curves are reliant to concentration of iron suspensions, and are resulting in convergence or divergence plots at pHs close to PZC [26, 27]. As found, PZC is independent on time contact and is corresponding to an extremum of pH against T variations. As discussed below, the relatively high point of zero charge value indicates the predominance of positively charged surface groups corresponding, in explored conditions, to $\overline{{ > {\text{F}}e{\text{OH}}}}$ [25].

### Potentiometric acid–base titration

The pH-dependent surface charge (Q) of iron (III) monohydrogen phosphate is studied by acid–base titration at μ value of 0.001, 0.01 and 0.1 M. Equilibrium titration is performed at ambient temperature for contact time (T) varying between 0.5 and 72 h.

The acid–base behavior of the phosphate surface is attributed to phosphohydrols ($\overline{{ > {\text{POH}}}}$) and ferrihydrols ($\overline{{ > {\text{FeOH}}}}$). The surface suspension acquires an electrostatic charge Q, owing to protonation/deprotonation processes that are well described as reactions of amphoteric ion exchanger materials ($\overline{{ > {\text{SOH}}}}$):

1 $$ \overline{{ > {\text{SOH}}}} {\text{ + H}}^{ + } \leftrightarrow  \overline{{ > {\text{S(OH}}_{2} )_{{}}^{ + } }} ,\quad {\text{K}}_{{{\text{eq}}}}^{ + } $$

2 $$ \overline{{ > {\text{SOH}}}} \leftrightarrow  \overline{{ > {\text{SO}}^{ - } }} {\text{ + H}}^{ + } ,{\text{ K}}_{{{\text{eq}}}}^{ - } $$

K_eq_^+^ and K_eq_^−^ respectively denote the surface stability constants, while the on the lined species belong to the solid phase. The pKa of $\overline{{ > {\text{POH}}}}$ and $\overline{{ > {\text{F}}e{\text{OH}}}}$ are respectively 1.44, and 4.66 [28, 29].

Generally, it is found that the particle surfaces of metal oxides are saturated and no more varying for pH ≤ 3 and pH ≥ 11 [30].

The synthesized iron phosphate is suspended in 25 ml of 0.05 M HNO_3_ and titrated with a standard solution of 0.05 M NaOH. The surface charge (Q) is determined using potentiometric techniques of time titration (PTT) and mass titration (PMT). In these methods, phosphate dispersions with different weights of 0.75, 1.25 and 2.5 g/L are titrated at contact time (T) that has varied between 0.5 and 72 h. A blank solution without iron phosphate is also titrated in these conditions.

### Surface charge of iron phosphate

The surface charge, Q, of phosphate suspension is obtained from curves titration obtained at given m, pH and T. The proton-reactive surface functional groups which are the main active elements in adsorption processes, are contributing also to acid–base properties of sorbent suspension. These properties which are the principal of PTT and PMT methods are based on pH difference between blank and phosphate suspension, for given neutralization volume of NaOH.

The charge Q is determined according to:

$$ Q = \frac{FC\Delta V}{m}\left( {{\text{Coulomb}}/{\text{g}}} \right) $$

The variations Q in function of pH allow determining both the point of zero charge (PZC) and isoelectric point (IEP).

F is the Faraday constant, C is the concentration of acid and base used in a titration, ∆V is the difference in the volume of base used to reach the same pH in dispersion and blank solution, and m (g/L) is the suspension concentration of the solid particles*.*

### pH-dependent surface charge in NaCl ectrolyte

**Fig. 6:**
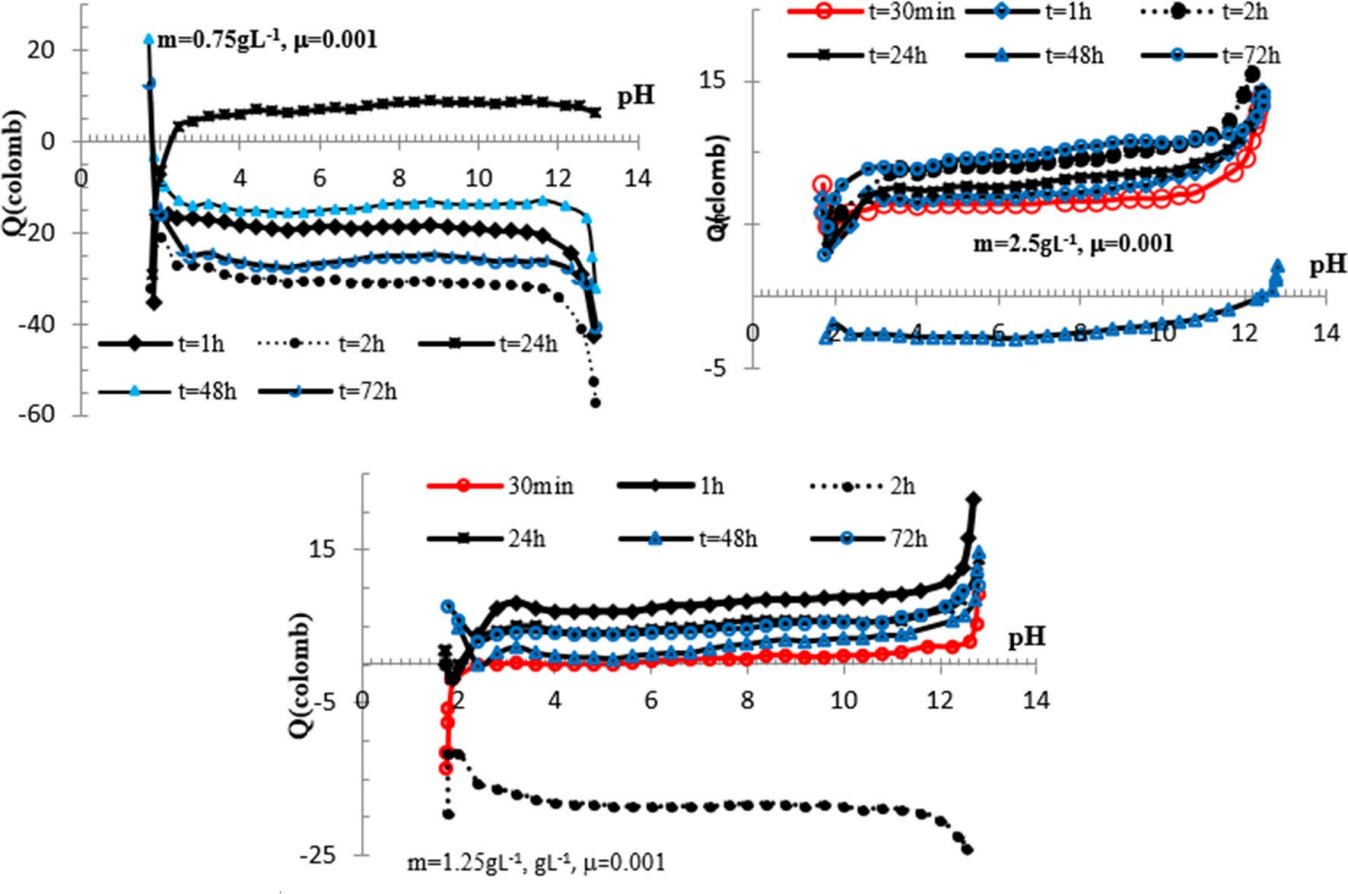
Variations Q in function of pH obtained for Fe_2_ (HPO_4_)_3_*4H_2_O in NaCl 0.001 M and m = 0.75, 1.25, 2.5 g L^−1^, $0.5\;{\text{h}} \le T \le 72\;{\text{h}}$

To study the pH-dependence of surface charge of phosphate particles, the Q value is determined at various μ and contact time (T). Figure 6 shows Q in function of T variations achieved at 0.001 M NaCl for phosphate suspensions (m) of 0.75, 2.15 and 2.5 g L^−1.^

During the suspension process, the iron phosphate surface develops a charge consequent to the Na^+^ or Cl^−^ insertion. This charge is contact time dependent while the permanent charged sites are involved in H^+^ (OH^−^)-exchange reaction. The negative charge on phosphate particles associated to 0.75 g L^−1^ is prevailing over the whole pH range. Q value ranging from − 27 to − 31 Coulombs is achieved in the pH range: 4.4–11.6, while a charge around − 18.5 coulombs is obtained in this condition at m = 1.25 g L^−1^. The positive charge obtained in the other explored conditions is no significant.

The absence of common intersection point of Q in function of pH curves shows that the characteristic pH of isoelectric point is dependent on contact time and mass suspension. The common intersection point of surface-charging curves observed at pH value of 1.8 is corresponding to IEP. The point of zero charge which corresponds to cross point of these variations is observed at pH around 2.4.

**Fig. 7:**
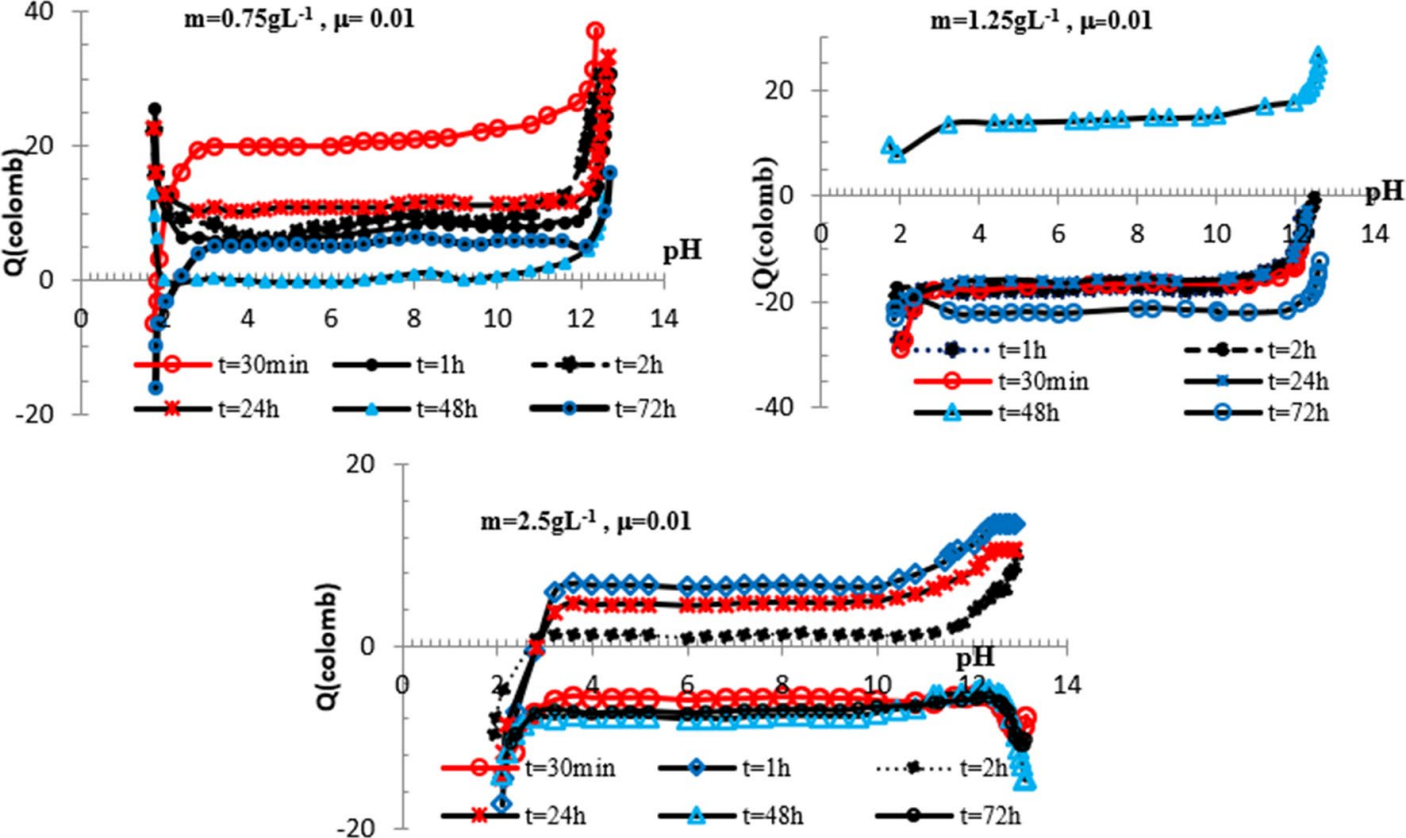
Variations Q in function of pH obtained for Fe_2_ (HPO_4_)_3_*4H_2_O in NaCl 0.01 M and m = 0.75, 1.25 and 2.5 g L^−1^,$0.5\;{\text{h}} \le T \le 72\;{\text{h}}$

**Fig. 8:**
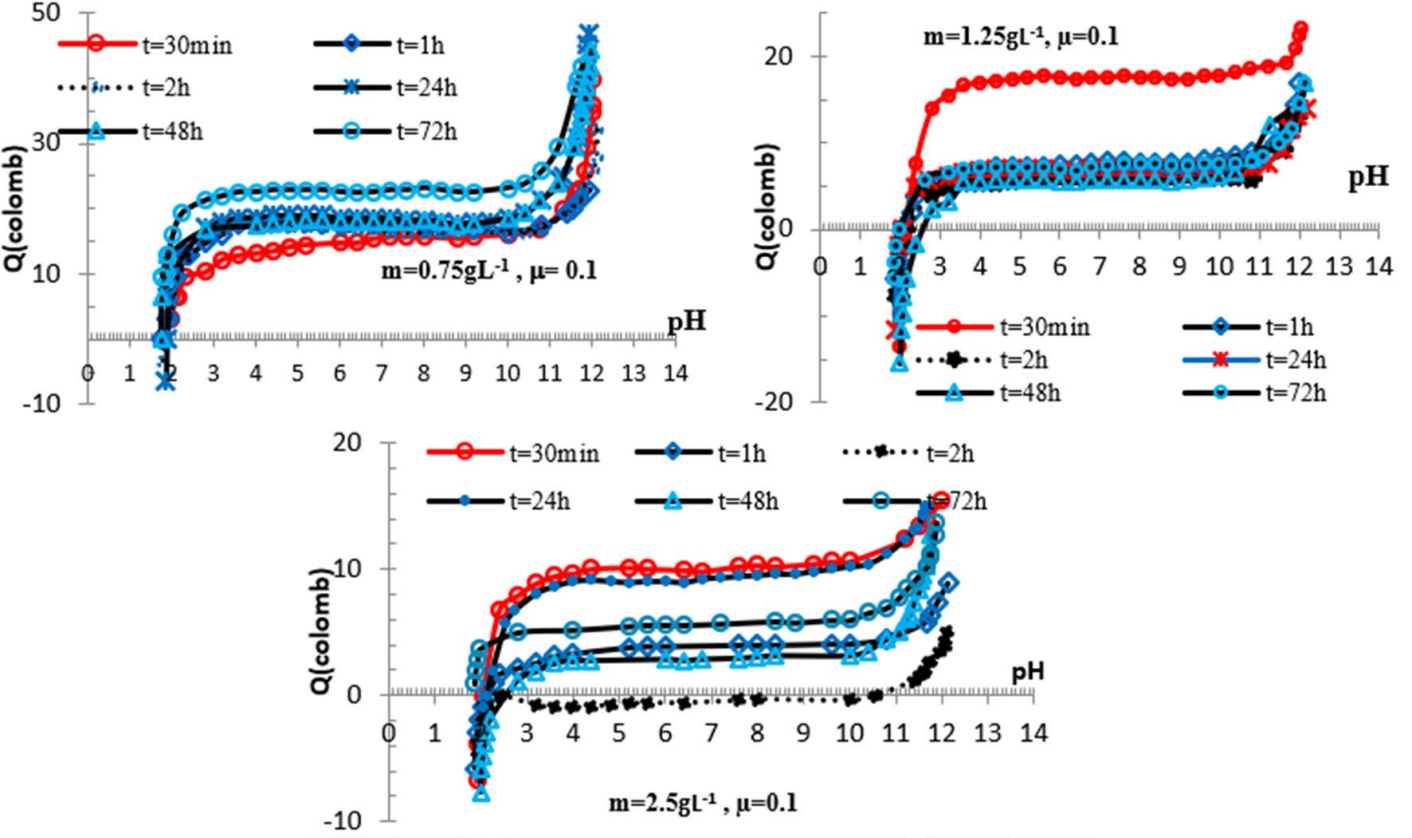
Variations Q in function of pH obtained at Fe_2_ (HPO_4_)_3_*4H_2_O in NaCl 0.1 M and m = 0.75, 1.25 and 2.5 g L^−1^, $0.5\;{\text{h}} \le T \le 72\;{\text{h}}$

The following curves in Figs. 7 and 8 show the variations Q in function of pH obtained for iron phosphate at 0.01 and 0.1 M NaCl.

As shown from 0.75 g L^−1^ suspension results, the IEP and the PZC are dependent on contact time and are respectively 1.8 and 2.4.

Figure 8 represents Q as a function of pH for different contact time.

Obtained results show that¸ as ionic strength increases, phosphate particles are no or less negatively charged. The maximum Q value is achieved at m = 0.75 g L^−1^ and µ = 0.1 for pH around 12. In this optimal pH conditions, the surface charge starts to become independent of contact time and increases rapidly to reach 50 coulombs. The similarity of Q against T curves with a positive plateau between pH 3 and 10.4, indicates that the increase in Q is essentially due to Na^+^ inclusion. A negative IEP is shown at pH around 1.8, while PZC value of 2.2 is achieved in these conditions. Moreover, at pH higher than PZC, a rapid shift of the surface charge toward positive values is occurred. It is important to note that the isoelectric point is comparable to PZC value of $\overline{{ > {\text{POH}}}}$ [28, 31].

### Surface complexation constant

The surface complexations involved in protonation/ deprotonation reactions are dependent on suspension acidity. As shown previously, the strength of surface functional groups are altered, due to inter- and intra-molecular interactions. Consequently, it is assumed that each definite set of individual $\overline{{ > {\text{SOH}}}}$ group is characterized by distinct acidity constants (K_a_) [32].

This result achieved at contact time of 24 h, is suggested to be due to the complexity and heterogeneity of sorbent surface. However, it is shown previously that sorption is more complexed kinetic phenomenon witch optimal conditions are not always corresponding to higher duration [5].

The results of potentiometric titrations carried out at various contact times show that the phosphate systems are characterized by comparable acidity constants, K_a_, that pK_a_ value is equal to 2.4 ± 0.2. One can note that this pK_a_ is equal to PZC of $\overline{{ > {\text{POH}}}}$ group which ionization seems to be rather correlated to the contact time than to nature of this surface acid group.

Taking this result into account, the surface charge behavior of synthesized Fe_2_ (HPO_4_)_3_*4H_2_O is dominated by the phosphohydrols $\overline{{ > {\text{POH}}}}$ which deprotonation reactions are prevailing under acidic conditions. The acidic sites are deprotonated following the reaction:

3 $$ \overline{{ > {\text{P(OH}}_{2} )_{{}}^{ + } }} \, \leftrightarrow \, \overline{{ > {\text{POH}}}} {\text{ + H}}^{ + } ,{\text{ pK}}_{{\text{a}}} $$

The experimental results show that pKa = 2.45 ± 0.15.

The partition equilibrium involving sodium ion insertion is:

4 $$ \, \overline{{{\text{ > P(OH}}_{{2}} {)}^{ + } }} {\text{ + Na}}^{ + } \, \leftrightarrow \, \overline{{ > {\text{P(OH}}_{{2}}^{ + } {\text{,Na}}^{ + } )}} {,} $$

$$ {\text{K}}_{{{\text{Na}}}} \approx \frac{Q}{{96500\overline{{\left[ { > {\text{SOH}}_{2}^{ + } } \right]^{ } }} \left[ {{\text{Na}}^{ + } } \right]_{0} }} $$

The overall surface complexation reaction is:

5 $$ \overline{{ > {\text{POH}}}} {\text{ + H}}^{ + } \, + {\text{ Na}}^{ + } \leftrightarrow \, \overline{{ > {\text{P(OH}}_{2}^{ + } ,{\text{Na}}^{ + } )}} {, } $$

$$ {\text{K}}_{{ > {\text{SOH}}}} \approx \frac{{{\text{K}}_{{{\text{Na}}}} }}{{{\text{K}}_{{\text{a}}} }} \, $$

Under optimal conditions corresponding to suspension of 0.75 g L^−1^ (0.067 M), μ = 0.1 and pH about 1.8, Q and $\overline{{\left[ { > {\text{SOH}}_{2}^{ + } } \right]^{ } }}$ are found to be equal respectively to 20C and 0.007 M. As a consequence log K_Na_ = − 0.68 ± 0.02 and logK_>SOH_ = 1.77 ± 0.27.

### Response surface analysis

**Fig. 9:**
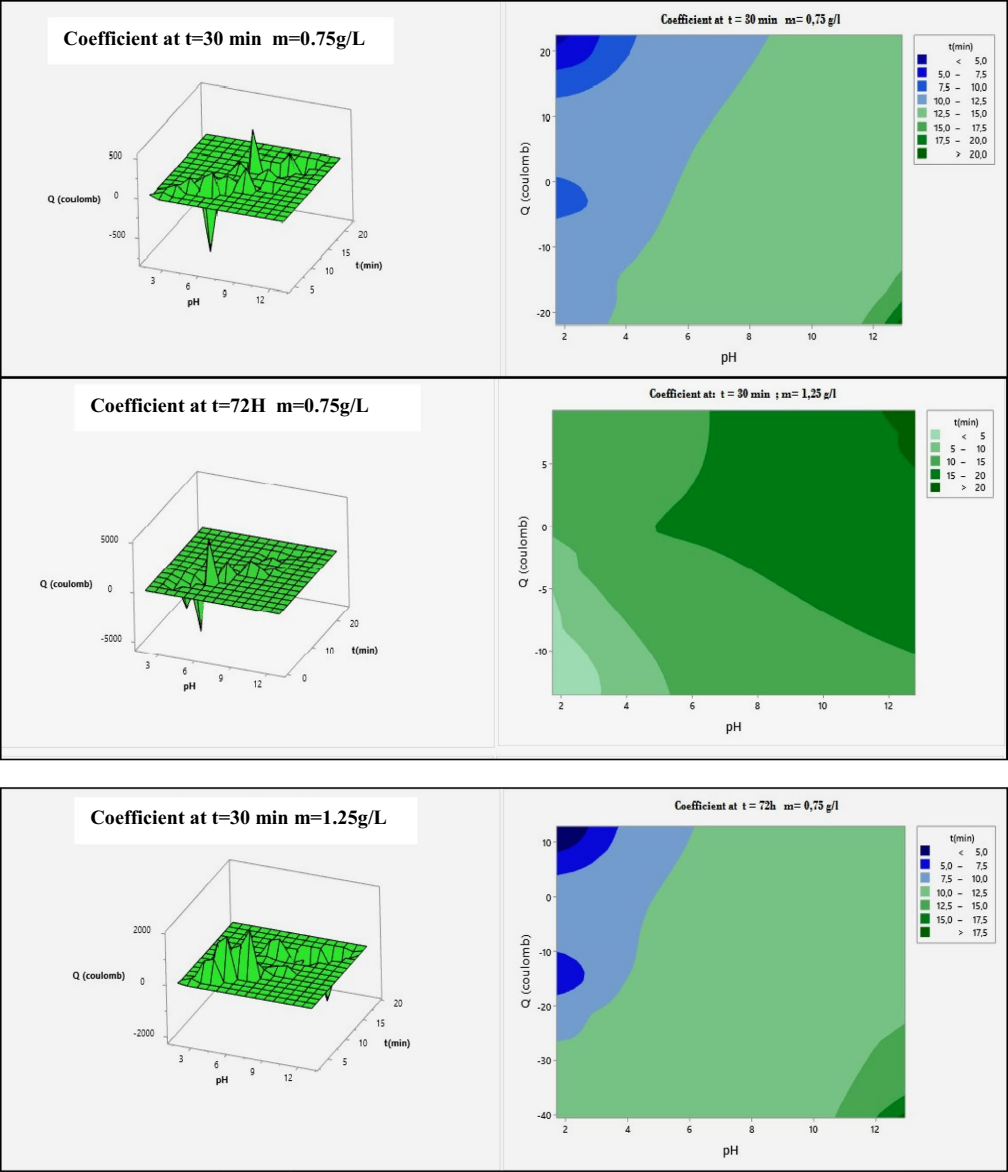

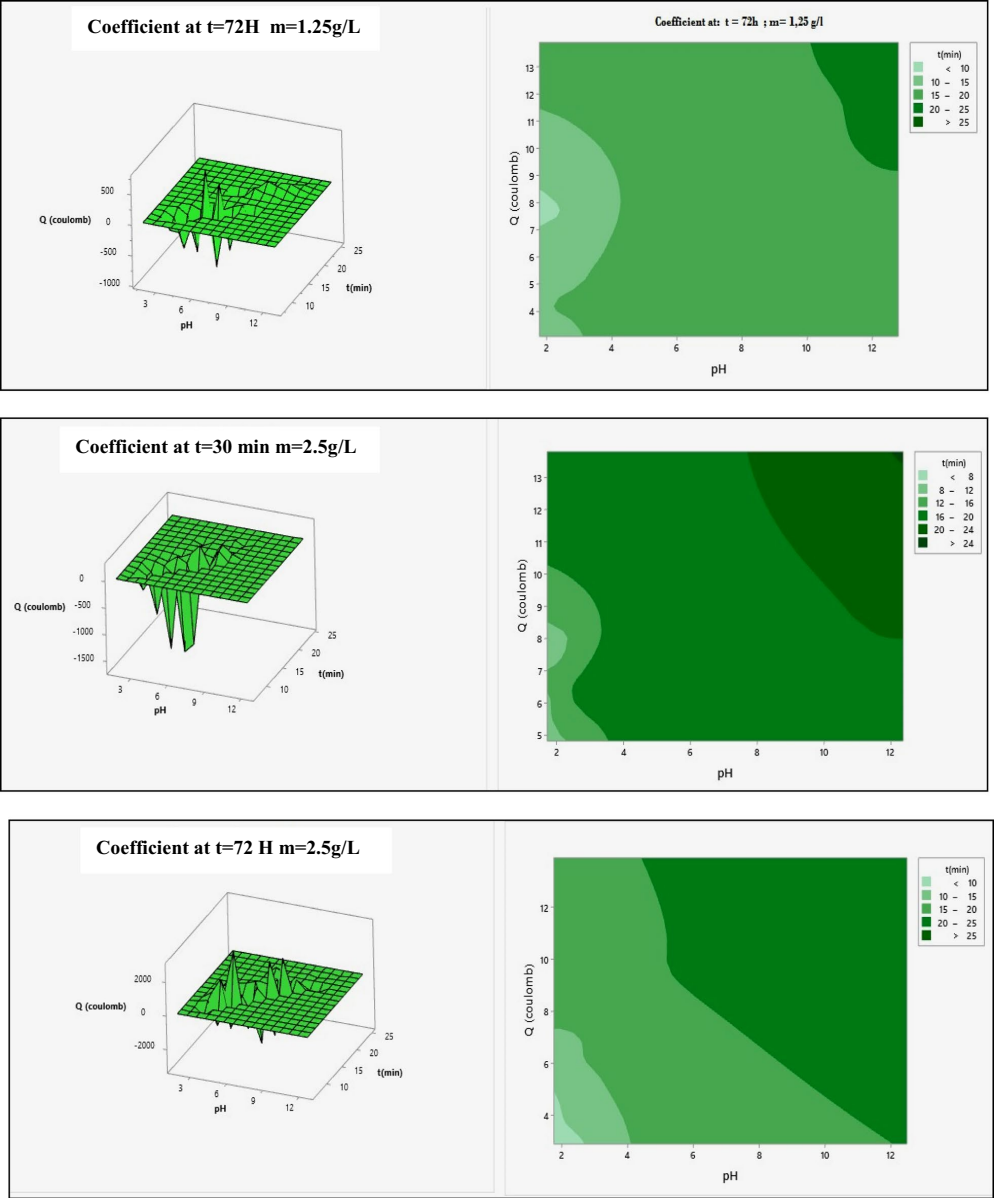

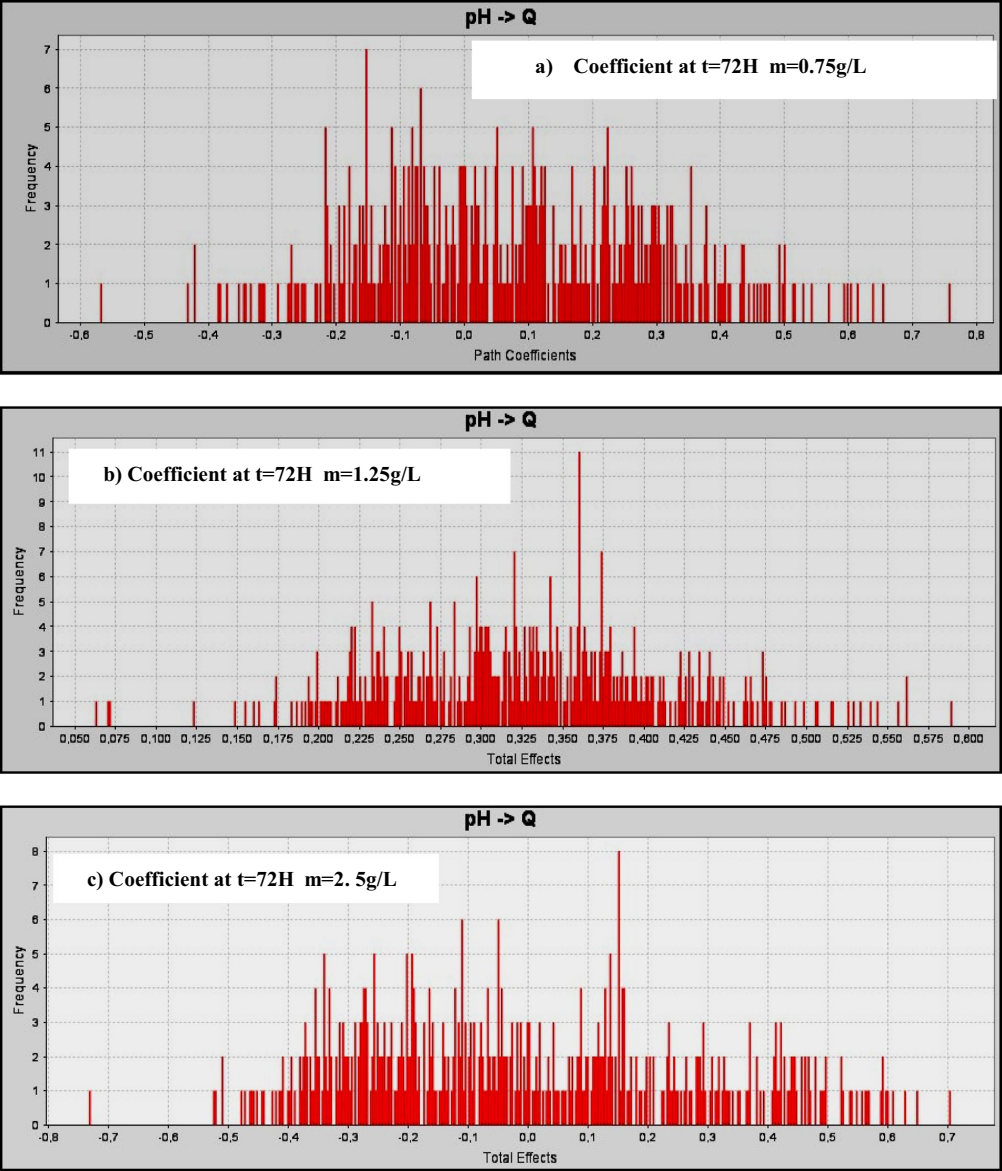
Surface response plots for the sodium insertion in amorphous Iron Phosphate for m = 0.75, 1.25 and 2.5 g L^−1^

The mathematical models for the for the sodium insertion in amorphous Iron Phosphate and dyes removal were used to build response surfaces as well as to determine the optimal conditions of the process. Figure 9 present the 3D response surfaces plots for the significant interactions.

The mathematical models for the sodium number were used to build response surfaces as well as to determine the optimal conditions of the process. Figure 9 presents the 3D response surfaces plots for the significant interactions.

For the sodium insertion number, the most significant interactions were the time, pH and Q. Figure 9 (m = 0.75 g/l) indicates that the sodium number increased with the increase of activation pH, Q and m ratio. Figure 9(m = 1.25 g/l) shows that the sodium insertion in amorphous Iron Phosphate increased with increase of the impregnation ratio and decrease of activation time when the activation time is fixed at 30 min.

For the t = 30 min and 72 h, m = 0.75 g/l index, the most significant interaction was the Q and activation pH. From Fig. 9(m = 1.25 g/l), it can be observed that the Sodium index increased with the increase of the activation Q and the pH. The maximal sodium index response was obtained at an activation time of 72 h.

For the t = 30 min and 72 h, m = 1, 25 g/l index, in the insertion of sodium, the same significant interactions are found, including the Q and pH. From Fig. 9(m = 2.5 g/l), it can be observed that the sodium insertion increased with increase of the activation time and Q. The maximal sodium responses were obtained at an activation time of 30 min. Figure 9(m = 2.5 g/l) shows that the sodium increased with decrease Q and decreased activation time in case the activation time is fixed at 72 h.

For the t = 30 min and 72 h, m = 2, 5 g/l index, the most significant interaction was the Q and activation pH. From Fig. 9(m = 1.25 g/l), it can be observed that the Sodium index increased with the increase of the activation Q and the pH. The maximal sodium index response was obtained at an activation time of 72 h.

## Conclusion

In the present study, the synthesis of iron (III) hydrogen phosphate is performed using inorganic sol–gel method coupled to microwave irradiation. The surface chemistry of obtained phosphate is performed using Kinetic-potentiometric method (KPM) which is a derived from the Potentiometric Mass Titrations (PMT) and Potentiometric Time Titrations (PTT) methods. The pH of common intersection of $\frac{{{\text{dpH}}}}{dT }\,in \,function\, of\, {\text{pH}}$ curves corresponds to PZC and IEP values that are found to be 4.3. Obtained results are used to evaluate the influence of synthesis conditions on the surface chemistry of the Fe_2_ (HPO_4_)_3_*4H_2_O. For these purpose, phosphate surface charge is studied at different ionic strength. The positive variable charge which is pH-independent is due to the accumulation of sodium ion in the material surface, mainly achieved by insertion process. The maximal surface charge (Q) is achieved at the low solid suspension. Hence, for m = 0.75 g L^−1^, Q value of 50 coulombs is carried at μ = 0.1 and pH around 12, while charge value around 22 coulombs is reached in the pH range: 3–10. These results are in agreement with those obtained previously indicating that the alkali ion diffusion in low suspensions medium is much faster than that in bulk. A shift is observed for PZC and IEP towards low values that are found to be 2.2 ± 0.2 and 1.8 ± 0.1, respectively. In acidic conditions, the surface charge behavior of synthesized phosphate is dominated by $\overline{{ > {\text{POH}}}}$ group which pKa = 2.45 ± 0.15. The equilibrium constant of sodium ion insertion is found to be log KNa = − 0.68 ± 0.02, while the overall surface complexation constant is logK > SOH = 1.77 ± 0.27.

## Data Availability

All data generated or analysed during this study are included in this published article.

## Abbreviations

PTT: Potentiometric Time Titration
PMT: Potentiometric Mass Titration
PZC: Point of zero charge
IEP: Isoelectric point
TGA: Thermogravimetric analysis
SEM: Scanning Electron Microscope
DTA: Differential thermal analysis
